# Effects of Interoceptive Awareness on Recognition of and Sensitivity to Emotions in Masked Facial Stimuli

**DOI:** 10.3390/bs15111555

**Published:** 2025-11-14

**Authors:** Kaho Yamasaki, Hiromitsu Miyata

**Affiliations:** 1 Graduate School of Letters, Arts and Sciences, Waseda University, Tokyo 162-8644, Japan; 2 Faculty of Letters, Arts and Sciences, Waseda University, Tokyo 162-8644, Japan; miyata@waseda.jp

**Keywords:** interoceptive awareness, mask, emotion recognition, valence, arousal

## Abstract

The present study examined associations between presence/absence of a mask and facial emotion recognition, and how interoceptive awareness, i.e., the perception of internal bodily sensations, may influence associations between them. Eighty-two university students participated in an online behavioral experiment. Participants were required to evaluate categories of emotions as well as valence and arousal levels of facial stimuli that were either neutral or expressed one of Paul Ekman’s basic emotions, i.e., anger, disgust, fear, happiness, sadness, and surprise. Participants also completed a psychological scale on interoceptive awareness. Results showed that accuracy of categorization was significantly lower and levels of valence and arousal were significantly closer to neutral in masked than in unmasked faces for multiple emotions. In addition, individuals who showed higher, as compared to lower, emotional awareness reported significantly higher levels of valence for masked stimuli that expressed surprise. These results suggest that wearing a mask can impair accuracy of facial emotion recognition and sensitivity to emotions, whereas awareness of the association between interoception and emotion might mitigate impairments of sensitivity to emotions in masked faces.

## 1. Introduction

Since the outbreak of the COVID-19 pandemic in 2020, wearing face masks has become a key strategy for infection prevention. While masks offer benefits for physical health, they are also suggested to hinder accurate recognition of others’ emotional expressions. A growing body of evidence suggests that wearing a mask can impair one’s ability to categorize various emotional expressions correctly. In fact, multiple studies have reported that masked stimuli that expressed neutral emotion and five of the six basic emotions except for anger (i.e., disgust, fear, happiness, sadness, and surprise) were more frequently misidentified compared to unmasked stimuli (Carbon, 2020; Ekman & Friesen, 1971; Shepherd & Rippon, 2022; Wong & Estudillo, 2022). Even before the pandemic, some studies examined the effect of limiting facial information by presenting only part of the face, such as the eye region (Han et al., 2012; Oliver et al., 2014) and/or one half of the face (Anzellotti & Caramazza, 2016). For example, Greening et al. (2018) found that expressions of disgust and happiness were less accurately recognized when only the eye region was visible compared to when the whole face was shown. In this context, studies have further investigated how accurately people can distinguish among different emotional expressions, especially when one expression is confused for another expression. Such studies reported that fear was frequently confused with surprise, and that disgust was evidently confused with anger when the lower half of the face was covered with a mask (Carbon, 2020; Rinck et al., 2022).

Wearing a mask has also been shown to impair the ability to accurately infer emotional intensity, which corresponds to arousal levels (Kastendieck et al., 2022; Levitan et al., 2022; McCrackin & Ristic, 2022; Tsantani et al., 2022). Russell and Bullock (1986) proposed the circular theory in which various types of emotions are mapped onto the coordinates on the axes of valence (i.e., pleasure–displeasure) and arousal (i.e., arousal–sleepiness; see also Russell et al., 1989; Russell & Lemay, 2000). Based on the circular theory, Tsantani et al. (2022) found that observers were prone to rate lower emotional intensity for masked facial stimuli expressing disgust, fear, happiness, sadness or surprise than for unmasked facial stimuli. Similarly, Kastendieck et al. (2022) reported that happy and sad faces were rated lower in intensity when those faces were masked than when they were unmasked. Levitan et al. (2022) also revealed that observers tended to report lower emotional intensity scores for positive emotions in both masked faces and the upper half of faces than in full faces. Furthermore, McCrackin and Ristic (2022) showed that masked facial stimuli that expressed happiness, neutral expressions, anger, and sadness were rated as less arousing and closer to neutral than unmasked facial stimuli. Harp et al. (2023) reported that masked faces expressing fear were less likely to be evaluated negatively, and masked faces expressing happiness were less likely to be evaluated positively, than unmasked facial stimuli. These findings suggest that wearing a mask can diminish emotional sensitivity, i.e., precision in the recognition of actual valence and arousal levels, to faces.

Given these impairments in accurate recognition of and sensitivity to facial expressions, it is essential to identify psychological and cognitive factors that may support accurate emotion recognition when external cues such as specific facial parts are restricted. Identifying such factors is crucial for facilitating smoother social communication under conditions such as widespread mask-wearing during a pandemic. One candidate is interoceptive awareness, the conscious perception of internal bodily sensations (Haruki et al., 2025). Predictive coding accounts provide a unifying framework that the brain uses to minimize prediction errors by integrating exteroceptive signals (e.g., facial expressions) with interoceptive signals (i.e., internal bodily states) to infer affective meaning (Barrett & Simmons, 2015; Seth & Friston, 2016). Within this framework, heightened interoceptive awareness may contribute to generating more accurate representations of internal bodily states, thereby compensating for reduced exteroceptive input when facial cues are masked. Given that interoception and exteroception share cognitive resources (Ren et al., 2024), sensitivity to internal signals may play a greater role in emotion recognition under such constraints than when exteroceptive information is fully available. At the psychological level, constructionist theories of emotion propose that emotions are constructed from core affect deriving from interoception (i.e., valence and arousal), and are shaped by knowledge and situational contexts (Barrett, 2006; Russell, 2003). From this perspective, interoceptive awareness may allow individuals to more accurately perceive the bodily feelings that underlie valence and arousal. Such sensitivity could, in turn, help them infer others’ emotions when facial cues are masked. Embodied emotion models further suggest that interoceptive brain regions such as the anterior insula and anterior cingulate cortex play a central role in monitoring bodily states and recognizing emotions in others (Craig, 2009; de Vignemont & Singer, 2006). Together, these perspectives converge on the idea that interoceptive awareness is a crucial factor for emotion recognition of others, particularly under conditions of reduced external emotional cues by a mask.

In line with this body of research highlighting the close relationship between interoception and emotion, several empirical studies have shown that interoceptive awareness is associated with the recognition of affective states of others (Critchley & Garfinkel, 2017; Price & Hooven, 2018). Terasawa et al. (2014) found that individuals with greater awareness of their own heartbeats, as indexed by higher accuracy in the heartbeat counting task, recognized anger, disgust, happiness, and sadness in facial expressions at lower emotional intensity levels than those with lower accuracy. Regarding interoceptive awareness assessed via self-report, Hübner et al. (2021) reported that individuals with higher interoceptive awareness in daily life showed lower thresholds for detecting emotions, which was reflected in shorter reaction times for emotional expressions at high intensity (see also Hübner et al., 2022). Ventura-Bort et al. (2021) found that higher self-reported interoceptive awareness was significantly associated with more accurate categorization of negative emotions (i.e., fear, anger, disgust, and sadness). Importantly, Imahuku et al. (2020) found that greater interoceptive awareness as reflected in heartbeat counting was associated with stronger facial mimicry when participants fixated on the eye region of facial stimuli. Although this study did not directly assess emotion recognition from eye region information alone, its findings suggest that interoceptive awareness may enhance sensitivity to emotional cues conveyed by the eyes. Considering these findings, it can be inferred that interoceptive awareness may facilitate the fine-grained recognition of emotional intensity and the accurate identification of discrete emotions when only the upper half of the face is visible.

Previous studies have suggested several factors that may influence emotion recognition in masked facial stimuli. McCrackin and Ristic (2022) reported that contextual information (e.g., her cat was found or lost yesterday afternoon) mitigated difficulty in recognizing valence and arousal in masked facial stimuli. Several studies also showed that social sensitivity, which is defined as the ability to perceive others’ mental states, predicted accuracy in recognizing emotions from masked facial expressions (Ikeda, 2024; Swain et al., 2022). Conversely, Maiorana et al. (2022) reported that higher tendencies for alexithymia, a psychological trait characterized by difficulties in recognizing and describing one’s own emotions (Sifneos, 1973), were associated with longer reaction times when categorizing emotions in facial stimuli with masked mouths or eyes. Despite these accumulating findings, the role of interoceptive awareness on recognition of and sensitivity to emotions in masked faces remains understudied.

Based on these underpinnings, the present study aimed to examine (1) associations between presence/absence of a mask and facial emotion recognition, and (2) potential effects of interoceptive awareness, on these associations. With respect to the first aim, we hypothesized that wearing a mask may hinder recognition of both categorization of emotions and levels of valence and arousal expressed in facial expressions. Specifically, we expected that accuracy in categorization of emotion would be lower for masked than for unmasked faces. Also, evaluation of valence and arousal levels were expected to be closer to neutral in masked than in unmasked faces. With respect to the second aim, We hypothesized that higher interoceptive awareness may be associated with more accurate and sensitive recognition of categories of emotion and arousal levels in masked faces. Specifically, we expected that, when presented with masked faces, individuals with higher self-reported interoceptive awareness in daily life, as measured by the Multidimensional Assessment of Interoceptive Awareness (MAIA; Mehling et al., 2012), may categorize emotions more accurately and report higher levels of valence and arousal than those with lower interoceptive awareness.

## 2. Methods

### 2.1. Participants

Eighty-two undergraduate and graduate students living in Japan (37 males and 43 females; sex was unknown for 2 individuals; 18–25 years; mean age = 20.37; *SD* = 2.72) participated in this study. All participants were recruited through the university’s online platform soliciting for part-time jobs. To participate in the study, participants were asked to access the URL for the experimental platform that was shown within the job recruitment. Prior to participation, participants read the objective and content of the study which were displayed on the browser. Participants gave informed consent by clicking a checkbox on the platform. After completing the study, participants received a reward of JPY 500 through bank transfer. Data collection was conducted on 10 and 11 October 2022.

### 2.2. Stimuli

Facial stimuli used for the present study were selected from the National Institute of Advanced Industrial Science and Technology (AIST) facial expression database 2017 (Fujimura & Umemura, 2018). The database contains 12 types of emotional expressions from eight Japanese models (four men and four women), i.e., 10 types of expressions with closed mouths (i.e., anger, disgust, fear, happiness, sadness, surprise, sleepiness, excitement, relaxation, and neutral) and 2 types of expressions with open mouths (i.e., anger and disgust). The facial stimuli were provided free of charge, under the condition that they were used only for research purposes and were not disclosed to external parties. For the test sessions, we used a total of 56 stimuli with closed mouths, each of which expressed either neutral emotions or one of the six basic emotions designated by Ekman and Friesen (1971), i.e., anger, disgust, fear, happiness, sadness, and surprise. For the practice session, we used eight stimuli with open mouths, all of which expressed anger, by selecting one stimulus from each model. All the selected stimuli were filmed from the front angle. Facial stimuli with a mask were virtually generated by superimposing an image of a nonwoven mask (*Matsukiyo mimi ga itaku narinikui mask*; Lib Laboratories Co., Ltd., Tokyo, Japan) over the lower half of the face by using Adobe Photoshop Elements 2019 (Adobe Inc., San Jose, CA, USA).

### 2.3. Psychological Scale

To assess sensibility of interoceptive awareness, we used a Japanese version of the Multidimensional Assessment of Interoceptive Awareness (MAIA) developed and validated by Shoji et al. (2018). The MAIA, originally developed by Mehling et al. (2012), is a widely used self-report scale that multidimensionally assesses awareness of interoceptive signals in daily life (Mehling et al., 2018). The Japanese version of the MAIA is composed of six subscales, each of which is designed to measure distinct facets of interoceptive awareness, i.e., *Noticing* (awareness of uncomfortable, comfortable, and neutral body sensations, 5 items: e.g., I notice how my body changes when I am angry.), *Not-distracting* (the tendency not to ignore or distract oneself from sensations of pain or discomfort, 3 items: e.g., I distract myself from sensations of discomfort.), *Attention Regulation* (the ability to sustain and control attention to bodily sensations, 7 items: e.g., I can refocus my attention from thinking to sensing my body), *Emotional Awareness* (awareness of a connection between body sensing and emotional states, 3 items: e.g., I notice that my breathing becomes free and easy when I feel comfortable.), *Body-listening* (the tendency to actively listen to the body for insight, 4 items: e.g., I listen to my body to inform me about what to do.), and *Trusting* (an experience of one’s body as safe and trustworthy, 3 items: e.g., I feel my body is a safe place.). Each item is rated on a six-point scale ranging from 0 (*Never*) to 5 (*Always*). Means of each subscale were used following the developers’ protocol, although hypotheses were formulated at the level of interoceptive awareness as a whole due to the lack of sufficient theoretical and empirical grounds and in accordance with conventions in previous studies (e.g., Atanasova et al., 2021; Calì et al., 2015). Internal consistency scores (Cronbach’s α) for each subscale in the present sample were 0.69, 0.78, 0.86, 0.86, 0.82, and 0.82, respectively.

### 2.4. Procedure

***General Procedure***. The present study was run entirely online using Gorilla Experiment Builder, a dedicated platform for online studies in psychology. Gorilla Experiment Builder is designed to minimize confounding factors stemming from variations in settings, equipment, and connection types. Its reliability as an experimental platform has been demonstrated by successful replications of experiments across diverse environments (Anwyl-Irvine et al., 2020). Participants were instructed to access the experimental platform from any location with internet access, by using either a laptop or a desktop computer equipped with a keyboard. They were not allowed to use a smartphone or a tablet. Participants were also instructed to use one of the following browsers: Firefox, Safari, Chrome, or Microsoft Edge, on their local computer. After the informed consent, the procedure for the experimental task was explained. After reading instructions of the procedure, participants underwent the practice session before proceeding to the test session. After the behavioral experiment, participants completed the MAIA scale by clicking checkboxes on the platform. Participation in the study took approximately 30 min in total.

**Figure 1:**
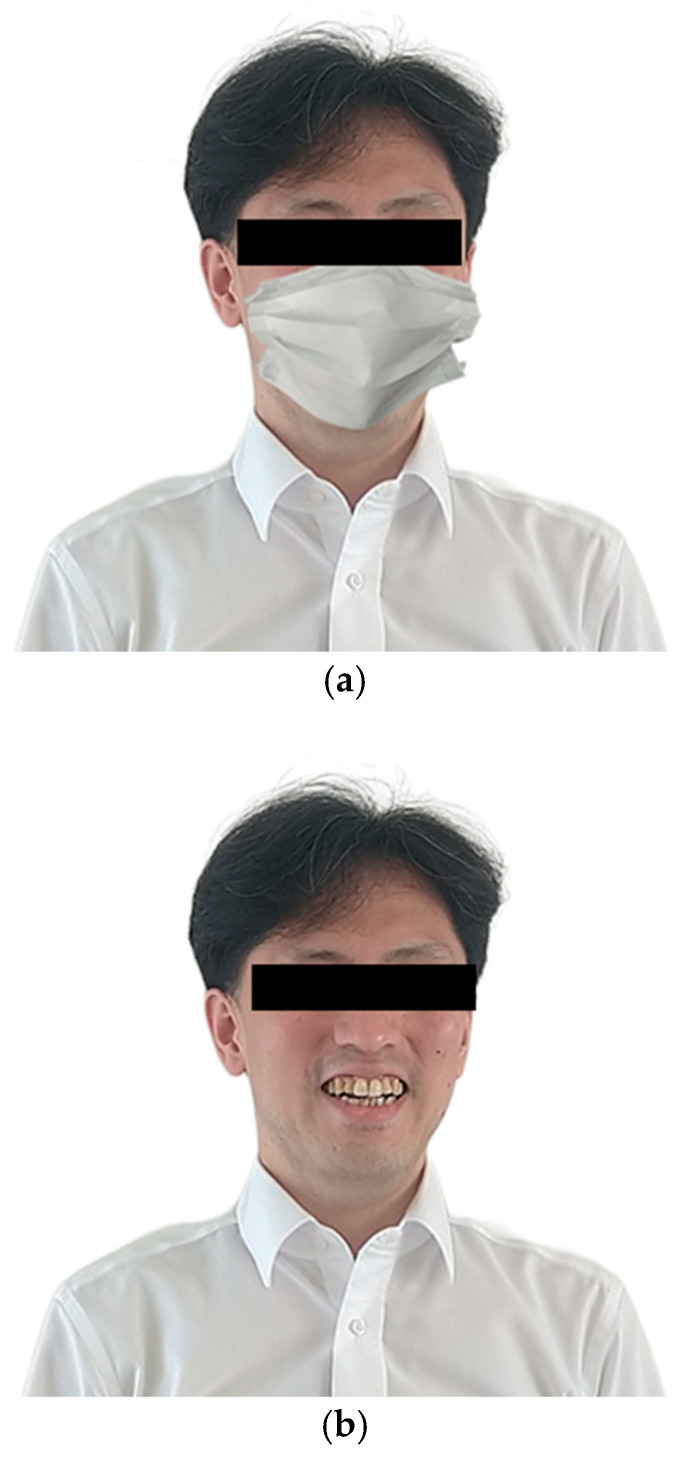
Examples of facial stimuli (**a**) with and (**b**) without a mask. Note that images shown in this figure are the examples created by the authors and were not actually used in the experiment. The eyes in the example images are covered with a black bar to prevent personal identification; black bars were not present in the actual experimental stimuli.

**Figure 2:**
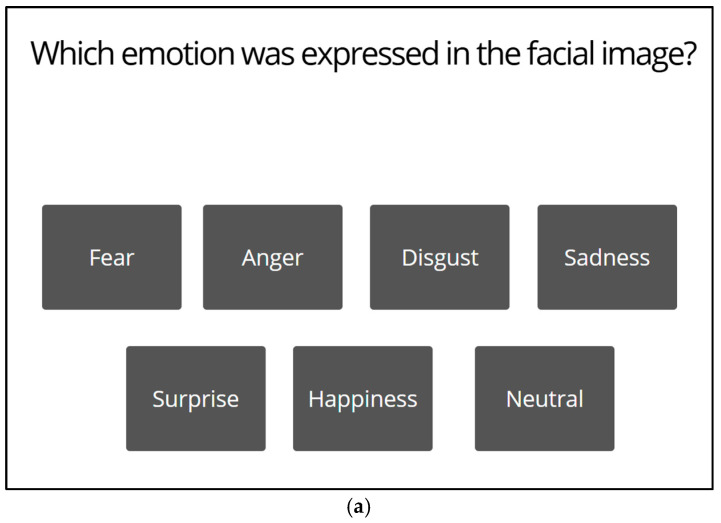

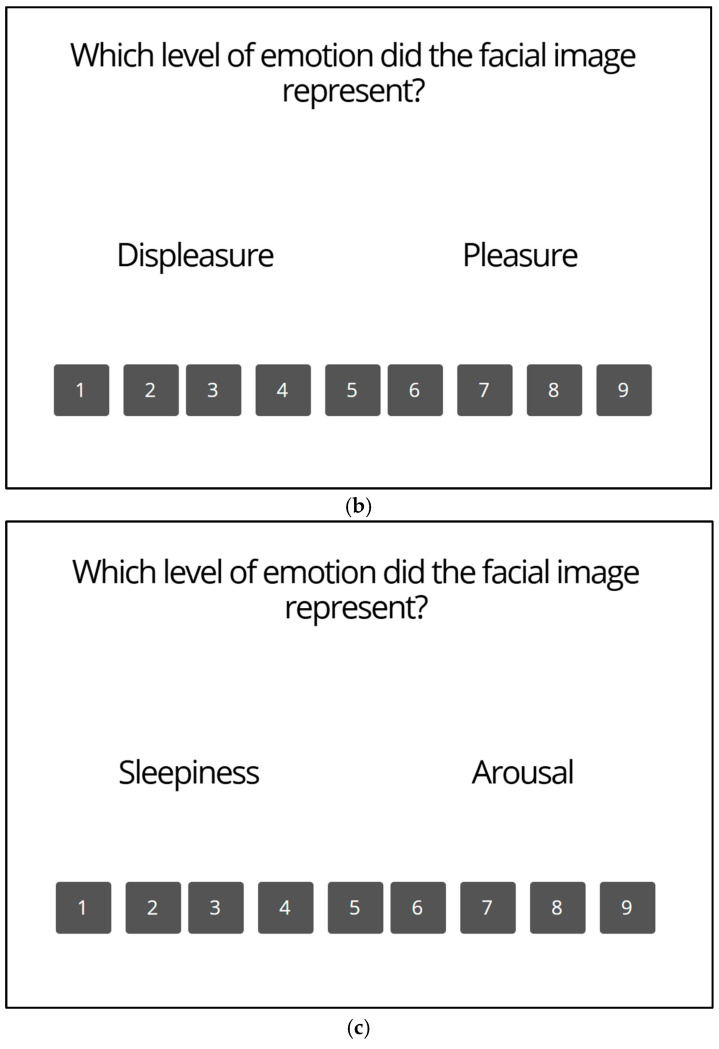
Examples of screens to require participants to evaluate (**a**) categories of emotions and levels of (**b**) valence and (**c**) arousal for each facial stimulus.

***Flow of each trial***. At the beginning of each trial, a fixation cross appeared at the center of the screen for 1 s. Following an interstimulus interval of 500 ms, a facial stimulus with a mask (Figure 1a) or with no mask (Figure 1b) was displayed on the screen for 2 s. Then, the next display for selection of the category of emotions appeared (Figure 2a). Participants indicated categorization of the emotional expression in each facial stimulus by clicking a relevant button presented on the display. Selecting one of the six basic emotions (excluding neutral) resulted in the next display, in which participants were required to evaluate valence of the facial stimulus (Figure 2b). Participants reported the valence of each face on a 9-point Likert scale (1 = *displeasure*; 9 = *pleasure*) by clicking a relevant button with a number on it. Next, the display appeared (Figure 2c), in which participants were required to rate arousal of the facial stimulus on a 9-point Likert scale (1 = *sleepiness*; 9 = *arousal*). Selection of the neutral option immediately resulted in the next trial without requiring participants to evaluate valence or arousal levels. There was no upper limit in the response times for these ratings.

***Practice and test sessions***. The practice session involved 8 (models) × 1 (emotion: anger with open mouths) × 2 (with or without a mask) = 16 trials. In the practice session, we used facial stimuli expressing only a single emotion (anger), so that they were consistent with those used in the test session. To prevent an anchor effect, we used facial stimuli with open mouths in the practice session. Each of the 16 stimuli were presented once in a pseudo-randomized order. After the practice session was completed, participants took an approximately 5-min break before proceeding to the test session. The test session generally replicated the procedure applied in the practice session. Specifically, the test session consisted of 8 (models) × 7 (emotions) × 2 (with or without a mask) = 112 trials. Each facial stimulus was presented once in a pseudo-randomized order. An approximately 5-min break was inserted after the participant completed 56 trials, so that the session was separated into two blocks.

### 2.5. Statistical Analysis Methods

Statistical analyses were conducted using a download-free software HAD 18 (Shimizu, 2016) and R (version 4.4.2). Prior to the main analyses, Cronbach’s α was calculated using R to assess the internal consistency of trial-by-trial scores in categorization, valence, and arousal. Cronbach’s α indicated acceptable internal consistency for categorization (α = 0.84), valence (α = 0.93), and arousal (α = 0.98) scores, suggesting that our tasks reliably measured the intended constructs across stimuli. Subsequently, means and standard deviations of accuracy in categorization of emotions for the mask or unmasked facial stimuli, valence and arousal levels for them, and the subscale scores from the MAIA were calculated. For valence and arousal levels, values averaged across the eight models were used as representative values for each participant. For scores from the subscales of the MAIA, median values were also calculated to categorize participants into groups based on the degrees of interoceptive awareness. Participants whose scores were below the median value were classified into the lower group, and those whose scores were the median value or above were classified into the higher group. Next, for accuracy of categorization of emotions (% correct) and levels of valence and arousal, respectively, we conducted three-way analyses of variance (ANOVA) using HAD 18 with presence of mask (mask or no-mask) and emotion (accuracy of categorization: seven emotions; valence and arousal: six emotions) as within-participants factors and interoceptive awareness (higher or lower groups based on scores from each subscale of the MAIA) as a between-participants factor. When a three-way interaction (mask × emotion × interoceptive awareness) was statistically significant, tests of simple interaction effects (e.g., whether the mask effect differed across emotions within higher vs. lower interoceptive awareness groups) were conducted. If a simple interaction was significant, we then examined simple–simple main effects, such as comparing masked versus unmasked faces for a particular emotion within each interoceptive awareness group, followed by post-hoc multiple comparisons. When a two-way interaction between any two factors (e.g., mask × emotion, mask × interoceptive awareness) was significant in the three-way ANOVA, we examined the corresponding simple main effects (e.g., the effect of mask within each emotion), again followed by post-hoc multiple comparisons. For post-hoc comparisons, *p*-values were not corrected because both the mask and interoceptive awareness factors consisted of only two conditions. Finally, to further examine the pattern of misclassifications, we constructed confusion matrices separately for the mask and no-mask conditions. For each cell of the matrix (true emotion × response), we computed the proportion of trials across participants. To determine whether the observed proportions significantly deviated from chance level, we conducted one-sample *t*-tests against the theoretical chance level (1/7 = 14.29%) for each off-diagonal cell (false alarms), as well as for the diagonal cells (hits). Holm corrections were applied within each true-emotion row to control for multiple comparisons (Greening et al., 2018).

## 3. Results

### 3.1. Descriptive Statistics

Means, *SD*s, and median scores for each subscale of the MAIA were as follows: 2.73 (*SD* = 0.89, median score = 2.60) for noticing, 2.55 (*SD* = 1.10, median score = 2.67) for not-distracting, 2.49 (*SD* = 0.91, median score = 2.43) for attention regulation, 2.53 (*SD* = 1.25, median score = 2.67) for emotional awareness, 2.15 (*SD* = 1.01, median score = 2.00) for body-listening, and 2.58 (*SD* = 1.11, median score = 2.67) for trusting. Table 1 displays the means and *SD*s for the accuracy of categorization, shown separately by mask condition (No-mask vs. Mask) and by groups split at the median on each MAIA subscale (Higher vs. Lower). Table 2 and Table 3 each show the rating scores for valence and arousal, presented for each mask condition (No-mask/Mask) and for each group based on MAIA subscale scores (Higher/Lower).

### 3.2. Results from Three-Way ANOVAs with Accuracy in Categorization of Emotions

- **Three-way ANOVAs**. Table 4 shows results from three-way ANOVAs with accuracy of categorization of emotions as a dependent variable. The three-way interaction (mask × emotion × interoceptive awareness) was statistically significant only for the analysis with the body-listening subscale from the MAIA as an independent variable. For the other MAIA subscales (i.e., noticing, not-distracting, attention regulation, emotional awareness and trusting), no significant three-way interaction was observed; however, two-way interactions between the presence of a mask and emotion were statistically significant. In addition, a two-way interaction between not-distracting and presence of mask was also significant. Regarding the main effects, the presence of a mask and emotion were significant, whereas interoceptive awareness was not significant across all MAIA subscales.
- **Post-hoc simple interaction analyses following a three-way interaction.** Because the three-way interaction involving body-listening was significant, post-hoc simple interaction analyses were conducted. These analyses revealed that simple interactions between presence of mask and body-listening were significant for the anger and disgust stimuli (anger: *F* [1, 560] = 8.385, *p* = 0.004, *ηp*^2^ = 0.095; disgust: *F* [1, 560] = 4.637, *p* = 0.032, *ηp*^2^ = 0.055). For fear, sadness, and surprise, only the main effect of the mask was significant (fear: *F* [1, 560] = 27.239, *p* < 0.001, *ηp*^2^ = 0.254; sadness: *F* [1, 560] = 55.904, *p* < 0.001, *ηp*^2^ = 0.411; surprise: *F* [1, 80] = 69.153, *p* < 0.001, *ηp*^2^ = 0.464). By contrast, statistically significant main effects or interactions were not observed for the happiness and neutral conditions.

For the mask condition, the simple interaction between body-listening and emotion (*F* [6, 960] = 2.963, *p* = 0.014, *ηp*^2^ = 0.036) and the main effect of emotion (*F* [6, 960] = 172.853, *p* < 0.001, *ηp*^2^ = 0.684) were significant. For the no-mask condition, only the main effect of emotion was significant (*F* [6, 960] = 138.734, *p* < 0.001, *ηp*^2^ = 0.634). Furthermore, simple interactions between mask and emotion were significant for both the lower body-listening group (*F* [6, 480] = 8.378, *p* < 0.001, *ηp*^2^ = 0.098) and the higher body-listening group (*F* [6, 480] = 9.557, *p* < 0.001, *ηp*^2^ = 0.088).

**Figure 3:**
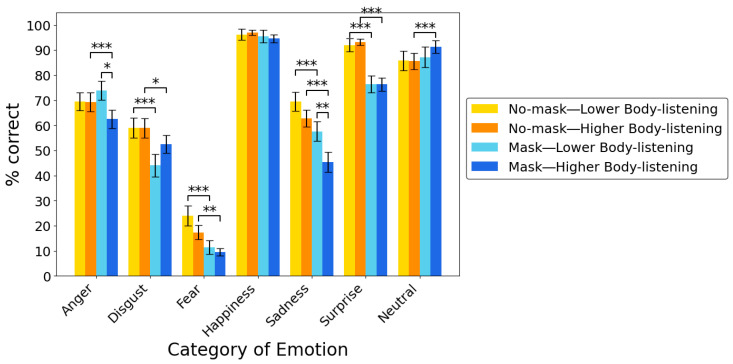
Bar plots for the mean accuracy of categorization of emotions, with groups divided based on the higher/lower scores from the body-listening subscale from the MAIA. Error bars indicate standard errors (*SE*s) of the mean. * *p* < 0.05, ** *p* < 0.01, *** *p* < 0.001.

- **Post-hoc multiple comparisons following simple interactions.** Given these significant simple interactions, post-hoc multiple comparisons were performed. Multiple comparisons further revealed that the accuracy for masked anger and sadness stimuli was significantly lower in individuals with higher body-listening than in those with lower body-listening (all *ps* < 0.05; Figure 3). Overall, these results show that individuals with a higher tendency to attend to interoception for guidance were less accurate in categorizing masked faces expressing negative emotions than those with a lower tendency to attend to interoception for insight.

Moreover, multiple comparisons revealed that accuracy of categorization of emotions was significantly lower in the mask condition than in the no-mask condition for disgust, fear, sadness, and surprise stimuli in the group with lower body-listening and for anger, disgust, fear, sadness, or surprise stimuli in the group with higher body-listening. By contrast, accuracy of categorization was significantly higher in the mask than in the no-mask condition for neutral stimuli in the group with higher body-listening (all *ps* < 0.001; Figure 3). Taken together, these results show that individuals with both lower and higher tendency to attend to interoception for insight exhibited lower accuracy in categorizing facial stimuli expressing disgust, fear, sadness, and surprise in the mask than in the no-mask condition. Also, individuals with higher tendency to attend to interoception for insight were more accurate in categorizing masked neutral expressions than those with lower tendency to attend to interoception for insight.

- **Post-hoc multiple comparisons following two-way interactions.** For the analyses with the noticing, not-distracting, attention regulation, emotional awareness, and trusting subscales from the MAIA as independent variables, multiple comparisons revealed that accuracy of categorization for stimuli expressing disgust, fear, sadness, and surprise was significantly lower in the mask than in the no-mask condition for noticing or attention regulation (all *ps* < 0.001). Similarly, for the analyses with not-distracting, emotional awareness, or trusting, accuracy of categorization for stimuli expressing disgust, fear, sadness, and surprise was significantly lower in the mask than in the no-mask condition (all *ps* < 0.05). In these analyses, however, accuracy of categorization for neutral stimuli was significantly higher in the mask than in the no-mask condition (all *ps* < 0.05). Collectively, these results show that accuracy of categorization for disgust, fear, sadness, and surprise stimuli was lower in the no-mask than in the mask condition. In contrast, accuracy of categorization for neutral stimuli was higher in the no-mask than in the mask condition.

Finally, multiple comparisons showed that for both the low and high not-distracting groups, accuracy of categorization was significantly lower in the mask than in the no-mask condition (all *p*s < 0.001). No statistically significant differences in the accuracy of categorization were observed between the high and low not-distracting groups in either the mask or no-mask conditions. These results show that accuracy of categorization was consistently lower in the mask condition than in the no-mask condition, independent of levels in not-distracting.

### 3.3. Results from Three-Way ANOVAs with Valence

- **Three-way ANOVAs.** Results from three-way ANOVAs with valence as a dependent variable are summarized in Table 5. A statistically significant three-way interaction was observed only for the analysis with the emotional awareness subscale from the MAIA as an independent variable. For the analyses involving the noticing, not-distracting, attention regulation, body-listening or trusting subscales from the MAIA as independent variables, two-way interactions between presence of mask and emotion were significant. The main effects of mask and emotion were statistically significant, whereas the main effects of interoceptive awareness were not significant for the all MAIA subscales.
- **Post-hoc simple interaction analyses following a three-way interaction.** Because the three-way interactions involving emotional awareness were significant, post-hoc interaction analyses were conducted. These analyses revealed significant simple interactions between presence of mask and emotional awareness for the happiness (*F* [1, 480] = 4.270, *p* = 0.039, *ηp*^2^ = 0.051) and surprise stimuli (*F* [1, 480] = 10.493, *p* = 0.0012, *ηp*^2^ = 0.116). For the disgust and fear stimuli, the main effect of presence of mask was significant in the simple interaction test that examined an interaction between presence of mask and emotional awareness (disgust: *F* [1, 480] = 54.215, *p* < 0.001, *ηp*^2^ = 0.404; fear: *F* [1, 480] = 62.670, *p* < 0.001, *ηp*^2^ = 0.439).

Simple interactions between emotional awareness and emotion were not significant for either the no-mask (*F* [5, 800] = 1.516, *p* = 0.220, *ηp*^2^ = 0.019) or mask (*F* [5, 800] = 0.755, *p* = 0.479, *ηp*^2^ = 0.009) conditions; however, the main effect of emotion was significant (no-mask: *F* [5, 800] = 478.700, *p* < 0.001, *ηp*^2^ = 0.857; mask: *F* [5, 800] = 347.672, *p* < 0.001, *ηp*^2^ = 0.813).

Furthermore, simple interactions between the presence of mask and emotion were significant for both the lower emotional awareness group (*F* [5, 400] = 18.790, *p* < 0.001, *ηp*^2^ = 0.155) and the higher emotional awareness group (*F* [5, 400] = 15.028, *p* < 0.001, *ηp*^2^ = 0.165).

**Figure 4:**
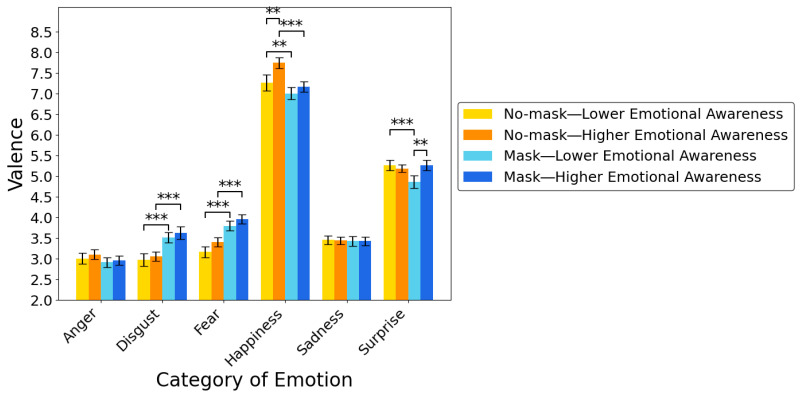
Bar plots for the mean rating scores of valence, with groups divided based on the higher/lower scores from the emotional awareness subscale from the MAIA. Error bars indicate standard errors (*SE*s) of the mean. ** *p* < 0.01, *** *p* < 0.001.

- **Post-hoc multiple comparisons following simple interactions.** Multiple comparisons further revealed that scores for valence were significantly lower in the mask than in the no-mask condition for disgust and fear stimuli in the groups with higher and lower emotional awareness (all *p*s < 0.001). In addition, scores for valence were significantly lower in the mask than in the no-mask condition for the happiness or surprise stimuli in the lower emotional awareness group, and for the happiness stimuli in the higher emotional awareness group (all *p*s < 0.001; Figure 4). Taken together, these results show that individuals with both low and high awareness of the link between interoception and emotion reported scores on valence that were closer to neutral for disgust, fear, and happiness stimuli in the mask than in the no-mask condition.

Moreover, multiple comparisons further revealed that, for facial stimuli with no mask that expressed happiness and for stimuli with a mask that expressed surprise, valence was significantly higher in the higher emotional awareness group than in the lower emotional awareness group (all *p*s < 0.01; Figure 4). These results show that, compared with individuals low in awareness of the link between interoception and emotion, those high in emotional awareness reported higher scores on valence on masked stimuli expressing happiness and unmasked stimuli expressing surprise.

- **Post-hoc multiple comparisons following two-way interactions.** As two-way interactions between mask and emotion were significant for the analyses with noticing, not-distracting, attention regulation, body-listening or trusting, we subsequently performed post-hoc multiple comparisons. Multiple comparisons following two-way interactions revealed that scores on valence for disgust and fear stimuli were significantly higher and scores on valence for happiness and surprise stimuli were significantly lower in the mask than in the no-mask condition (all *p*s < 0.05). These results show that scores on valence in masked stimuli expressing disgust, fear, sadness, and surprise were closer to neutral compared with those in unmasked stimuli.

### 3.4. Results from Three-Way ANOVAs with Arousal

- **Three-way ANOVAs.** Table 6 summarizes results from three-way ANOVAs with arousal as a dependent variable. A three-way interaction between presence of mask, emotion, and interoceptive awareness was statistically significant only for the analysis with the emotional awareness subscale from the MAIA as an independent variable. For the analyses with the noticing, not-distracting, attention regulation, body-listening and trusting subscales from the MAIA as independent variables, two-way interactions between presence of mask and emotion were significant. Main effects of mask and emotion were statistically significant. The main effect of interoceptive awareness was also significant for the analysis with attention regulation, showing that arousal scores were higher in the higher attention regulation group compared with the lower group.
- **Post-hoc simple interaction analyses following a three-way interaction.** Because the three-way interaction involving emotional awareness was significant, post-hoc simple interaction analyses were conducted. These analyses revealed a significant simple interaction between presence of mask and emotional awareness for the surprise stimuli (*F* [1, 480] = 7.435, *p* = 0.007, *ηp*^2^ = 0.085). Regarding the happiness and sadness stimuli, simple interaction test that involved an interaction between presence of mask and emotional awareness revealed a significant main effect of presence of mask (happiness: *F* [1, 480] = 15.267, *p* < 0.001, *ηp*^2^ = 0.160; sadness: *F* [1, 480] = 4.937, *p* = 0.027, *ηp*^2^ = 0.058), although no other significant main effects nor interactions were observed.

In contrast, simple interaction between emotional awareness and emotion was significant for neither the no-mask (*F* [5, 800] = 0.207, *p* = 0.915, *ηp*^2^ = 0.003) nor the mask conditions (*F* [5, 800] = 1.340, *p* = 0.258, *ηp*^2^ = 0.016).

Furthermore, simple interaction between presence of mask and emotion was significant in the lower emotional awareness group (*F* [5, 400] = 4.755, *p* = 0.004, *ηp*^2^ = 0.044), but not in the higher emotional awareness group (*F* [5, 400] = 2.673, *p* = 0.052, *ηp*^2^ = 0.034). In the higher emotional awareness group, only the main effect of emotion was significant (*F* [5, 400] = 26.512, *p* < 0.001, *ηp*^2^ = 0.438).

- **Post-hoc multiple comparisons following simple interactions.** Given these significant simple interactions, post-hoc multiple comparisons were performed. These comparisons revealed that scores on arousal were significantly higher for the sadness stimuli in the mask than in the no-mask condition in individuals with lower emotional awareness. Conversely, scores on arousal were significantly lower in the mask than in the no-mask condition for happiness stimuli in the lower emotional awareness group and for surprise stimuli in the higher emotional awareness group (*p*s < 0.001). These data show that scores on arousal for masked facial stimuli were lower than those on unmasked stimuli, regardless of perceived connection between interoception and emotion.

Multiple comparisons revealed no statistically significant differences in the scores on arousal for masked and unmasked stimuli between the higher and lower emotional awareness groups (all *p*s > 0.05).

- **Post-hoc multiple comparisons following two-way interactions.** As significant two-way interactions between mask and emotion were found for the analyses with the noticing, not-distracting, attention regulation, body-listening and trusting, we further examined them using post-hoc multiple comparisons. These analyses revealed that arousal was significantly lower for happiness and surprise stimuli, and significantly higher for sadness stimuli, in the mask than in the no-mask condition (all *p*s < 0.05). These data show that participants reported masked stimuli expressing sadness as being more aroused and masked stimuli expressing happiness or surprise as being less aroused than unmasked stimuli expressing these emotions.

### 3.5. Error Patterns in Categorization of Emotions

**Figure 5:**
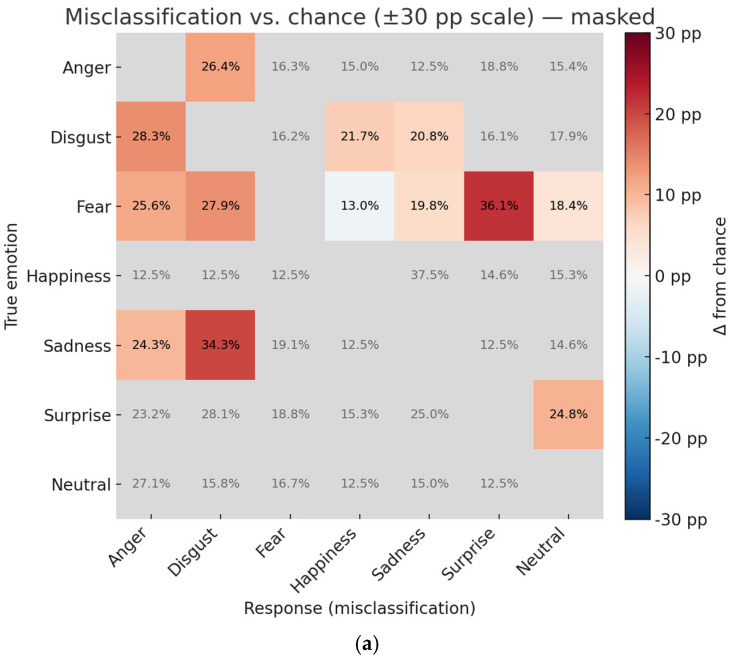

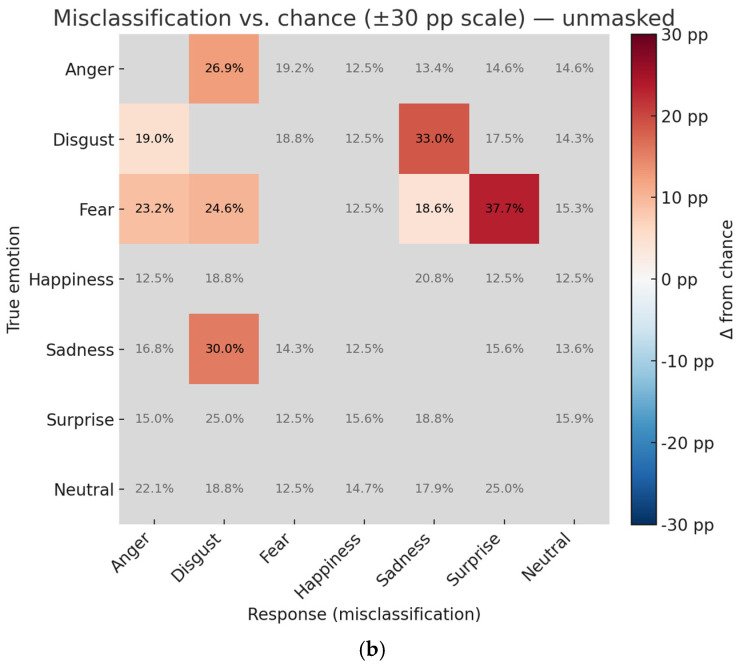
Misclassification matrices for (**a**) masked facial expressions and for (**b**) unmasked facial expressions. Correct responses (diagonal cells) are omitted. Cell colors indicate deviation from chance level (1/7 = 14.29%): red = significantly above chance level, blue = significantly below chance level, and gray = not significant after Holm correction for multiple comparisons. Numbers denote mean response proportions (%). The color scale is fixed to ±30 percentage points (*pp*s) for comparability across panels.

Figure 5 illustrates the misclassification matrices for (a) masked and (b) unmasked facial expressions, with correct responses (diagonal cells) omitted. Cells in red indicate response proportions significantly greater than chance level, whereas blue cells indicate those significantly lower than chance level, after Holm correction for multiple comparisons.

For masked faces, fear stimuli were frequently misidentified as surprise (*t*(79) = 12.45, *adjp* < 0.001, *d* = 1.39), and surprise stimuli were often misidentified as fear (*t*(79) = 10.83, *adjp* < 0.001, *d* = 1.21). In addition, sadness stimuli tended to be categorized as disgust more often than expected (*t*(79) = 7.02, *adjp* < 0.001, *d* = 0.79). Conversely, some confusions (e.g., happiness with sadness) occurred significantly less frequently than chance level (*t*(79) = −6.88, *adjp* < 0.001, *d* = −0.77).

For unmasked faces, overall categorization was closer to chance expectations, and misclassification patterns were less systematic. Nevertheless, a consistent tendency remained for fear stimuli to be misidentified as surprise and vice versa, although the magnitude of these effects was smaller than in the masked condition.

These results show that the presence of a mask amplifies systematic misclassification patterns, particularly among negatively valenced emotions such as fear, surprise, and sadness.

## 4. Discussion

The present study examined accuracy in categorization of emotions and sensitivity of emotion recognition in masked faces, as well as potential impacts of interoceptive awareness on emotion recognition in masked facial stimuli. Overall, the results suggest that emotions expressed in the facial stimuli with masks were less accurately identified, and that they were perceived as closer to neutral for both valence and arousal dimensions compared with emotions expressed in faces with no masks. Interestingly, interoceptive awareness was found to have differential effects on emotion recognition, depending on the dependent variables. Specifically, individuals with a higher emotional awareness dimension of interoceptive awareness perceived masked faces that expressed surprise as higher in valence than those with lower emotional awareness did. On the other hand, individuals with lower body-listening dimension of interoceptive awareness were more accurate in categorizing masked faces expressing disgust and fear than those with higher body-listening.

### 4.1. Effects of a Mask on Recognition of and Sensitivity to Emotions

Consistent with preceding studies on mask-wearing (Carbon, 2020; Rinck et al., 2022; Shepherd & Rippon, 2022; Wong & Estudillo, 2022) and broader research on partial-face recognition (Anzellotti & Caramazza, 2016; Greening et al., 2018; Han et al., 2012; Oliver et al., 2014), results from the present study support the notion that wearing facial masks can impair the accurate recognition of multiple emotions, including anger, sadness, fear, and surprise. Exploratory confusion-matrix analyses further revealed that errors were systematic rather than random: fear was frequently misclassified as surprise, and disgust as anger. Such misidentification patterns converge with findings by Greening et al. (2018), who showed that partial occlusion of the face induces non-random confusions between expressions with overlapping visual features. From the perspective of the Facial Action Coding System (Ekman & Friesen, 1978; Keltner et al., 2019), these confusions are consistent with overlapping Action Units (AUs) in the upper face, such as the eyebrows and eyelids. Specifically, both anger and fear involve raising the upper lids (AU 5) and lowering the eyebrows (AU 4) components. Fear and surprise share raising the inner eyebrows (AU 1), raising the outer eyebrows (AU 2), and raising the upper lids (AU 5). Consistently, both sadness and fear involve raising the inner eyebrow (AU 1) and lowering the eyebrows (AU 4). The present study required participants to judge categories of emotions based on these emotional cues around the eyes, because cues in other facial parts were occluded by masks. Therefore, wearing a mask may have hindered the ability to distinguish these emotional expressions (Rinck et al., 2022). In contrast, no statistically significant difference was found in the accuracy to identify happiness between the mask and no-mask conditions. This may be because happiness uniquely involves the Duchenne smile, which refers to a genuine smile characterized by wrinkles around the eyes arising from raising the cheeks (AU 6), which are not occluded by a mask (Gunnery & Ruben, 2015; Keltner et al., 2019). The present study also found that neutral stimuli were categorized more accurately in the mask than in the no-mask condition. This could be attributed to the fact that neutral faces with no masks appear to give slightly negative impressions (Lee et al., 2008). Thus, partial occlusion of emotional cues induced by wearing a mask may have resulted in the neutral facial expressions being interpreted as more neutral in the present study.

The presence of a mask was found to make participants’ evaluation of valence for disgust, fear, surprise, and happiness closer to neutral. These findings suggest that a facial mask may introduce ambiguity in the perception of valence. Previous studies on emotion recognition of masked faces primarily focused on whether masked facial stimuli are judged to convey pleasant or unpleasant emotional valence (e.g., Harp et al., 2023). The present study extends this line of research by providing novel evidence of reduced perceived intensity of valence across multiple emotions. Consistent with previous studies (Kastendieck et al., 2022; Levitan et al., 2022; McCrackin & Ristic, 2022; Tsantani et al., 2022), we also found that the presence of a facial mask made participants’ evaluation of arousal for faces expressing sadness, happiness, and surprise closer to neutral. Evaluations of valence and arousal are thought to rely on salient intensity-related cues specific to each emotion: the smiling mouth conveys positive valence in happiness, the distorted mouth conveys negative valence in disgust, and the downturned mouth corners provide a low-arousal cue in sadness (Calvo & Nummenmaa, 2008, 2016; Beaudry et al., 2014; Scherer & Ellgring, 2007; Keltner et al., 2019). For fear, wide-open eyes provide a strong cue, but negative valence evaluations typically rely on the combined saliency of both the eyes and the open mouth (J. W. Tanaka et al., 2012; Calvo et al., 2012). Thus, when the mouth is occluded by a mask, the remaining eye information may be insufficient to support a clear negative valence judgment. In addition, wide-open eyes and an open mouth contribute both to valence and arousal judgments in surprise. Mask occlusion removes many of these diagnostic cues from the lower part of the face, thereby eliminating crucial sources of information for both valence and arousal. This likely explains why participants’ evaluations of valence and arousal were shifted toward neutral in the present study.

### 4.2. Effects of Interoceptive Awareness

Individuals with higher scores on body-listening, which is characterized by sustained attention to bodily sensations, exhibited lower accuracy in categorizing masked stimuli compared to those with lower scores on body-listening. Although our hypothesis concerned interoceptive awareness as a whole, this subscale-level effect emerged in the analyses and therefore should be interpreted with caution. A potential reason for these results concerns the fact that the present study design assumed multisensory processing of visual and interoceptive signals. Y. Tanaka et al. (2023) provided insights into allocation of attentional resources in multisensory perception, using an auditory oddball task synchronized with heartbeat by using heartbeat-evoked potentials (HEP) and event-related potentials (ERP). Amplitudes of both HEP (reflecting interoception) and ERP (reflecting auditory processing) were largest when pure tones were presented during diastole. Tanaka et al. suggest that interoceptive and auditory stimuli share common attentional resources when they were processed simultaneously. Assuming that these findings may extend to simultaneous processing of interoceptive and visual stimuli, individuals high in body-listening may have directed a relatively large amount of attentional resources to their own interoceptive signals when presented with the facial stimuli. Consequently, individuals high in body-listening may have allocated relatively fewer attentional resources to facial expressions, thus reducing their ability to recognize visual cues required to accurately categorize emotions.

Regarding evaluation of valence, individuals high in emotional awareness, i.e., awareness of the connection between bodily sensations and emotional states, perceived masked faces expressing surprise as more pleasant than those low in emotional awareness. This result partially supports the view that interoceptive awareness may help compensate for the evaluation of valence when facial emotional cues are restricted by a mask. From a predictive coding perspective, interoceptive awareness may refine internal bodily representations and thereby strengthen valence judgments when exteroceptive information is reduced (Barrett & Simmons, 2015; Seth & Friston, 2016). Constructionist and embodied emotion models similarly emphasize the role of interoceptive processes in shaping core affective dimensions and supporting recognition of others’ emotions (Barrett, 2006; Russell, 2003; Craig, 2009; de Vignemont & Singer, 2006). Importantly, this finding provides novel evidence that a specific aspect of interoceptive awareness can enhance the evaluation of valence in masked facial expressions. Although this effect was limited to one subscale and one emotional category, it suggests that interoceptive awareness may serve as a psychological resource supporting emotional sensitivity when facial cues are partially obscured. More broadly, this finding contributes to understanding how interoceptive awareness may facilitate social cognition under constrained communicative contexts, such as mask-wearing during pandemics or in clinical settings.

On the other hand, interoceptive awareness did not significantly influence participants’ evaluation of arousal for masked faces. This might be due to the characteristics of the psychological scale used in the present study. Other than the MAIA, several psychological scales exist for measuring interoceptive awareness. For example, both the Autonomic Perception Questionnaire (Mandler et al., 1958) and the Body Perception Questionnaire (Porges, 1993) measure frequency of experience of specific sensations that arise from autonomic nervous activities and/or internal organs. Considering the fact that both interoception and emotion are suggested to arise from perception of specific autonomic and somatic activities (Barrett et al., 2004; Russell & Barrett, 1999), subjective experience of specific interoceptive sensations may be more closely related to perception of arousal in facial expressions than to interoceptive awareness in daily life. Future research may examine whether and how subjective evaluation of interoceptive awareness might influence perception of arousal in masked facial expressions.

Taken together, the present findings provide preliminary but meaningful evidence that aspects of interoceptive awareness can differentially shape emotion recognition when facial cues are restricted by a mask. Theoretically, these results extend predictive coding, constructionist, and embodied accounts by showing that interoceptive awareness not only refines internal affective representations but also interacts with attentional allocation to influence sensitivity to others’ emotions. Practically, the present study highlights interoceptive awareness as a potential psychological resource that could be targeted to support social communication under constrained conditions, such as widespread mask-wearing during pandemics or in clinical populations where facial expressivity is reduced. Although the observed effects were limited in scope and require replication, the present study contributes to bridging the gap between interoception research and applied challenges in everyday social interaction. In particular, interoceptive interventions that enhance awareness of internal bodily signals (e.g., meditation and yoga; Weng et al., 2021) may offer promising avenues for fostering sensitivity to others’ emotions under conditions where facial cues are constrained, such as during mask-wearing.

### 4.3. Limitations and Future Perspectives

Despite these novel findings and perspectives, the present study has apparent limitations. Firstly, control over the experimental environment was inevitably limited because the present study was entirely conducted online. Although the Gorilla platform has been validated to provide reliable stimulus timing across browsers and operating systems (Anwyl-Irvine et al., 2020; Bridges et al., 2020), online experiments are inevitably accompanied by methodological constraints. A major issue is the lack of experimental control (Grootswagers, 2020). For example, it is impossible to regulate participants’ viewing distance, posture, location, or ambient environment, making precise control of stimulus perception difficult. Moreover, participants’ concentration and motivation cannot be directly monitored, and they may be distracted by other sources (e.g., mobile phones, conversations, background noise), which introduces uncontrolled variance in performance (Grootswagers, 2020; Sauter et al., 2020). Although these uncontrolled factors are unlikely to have systematically distorted the observed effects, they may have added random noise to the data and thus reduced the statistical sensitivity of the present study. Future research conducted under laboratory settings, where the testing environment can be rigidly controlled, would help to confirm the robustness of the present findings.

The second limitation relates to the relatively low ecological validity of the facial stimuli. The present study used images with digitally overlaid masks to maintain strict control of facial features. Although this method ensured consistency across conditions, it may have reduced ecological validity. Future research should therefore employ photographs of individuals actually wearing masks to enhance the generalizability of the findings to real-world social interactions.

The third limitation concerns potential individual difference variables that were not directly measured in this study. Previous studies have shown that alexithymia is moderately associated with difficulties in emotion recognition (e.g., Bird & Cook, 2013; Grynberg et al., 2012) and is closely related to low interoceptive awareness (e.g., Murphy et al., 2018; Brewer et al., 2016). Therefore, it remains possible that the effects observed here partly reflect the influence of alexithymia rather than interoceptive awareness alone. Future research should measure both constructs simultaneously to disentangle their unique and overlapping contributions to emotion recognition. In addition, familiarity with masked faces represents a distinct individual difference factor. Previous studies have suggested that prior experience with masked faces may shape performance in emotion recognition (Ross & George, 2022; Karmakar & Das, 2023). In Japan during 2022, the use of masks in daily life was widespread, which likely minimized individual variability in familiarity with masked faces within our sample. Nevertheless, in other cultural or temporal contexts, such familiarity may emerge as an important factor influencing performance in emotion categorization and ratings of valence and arousal. Future research should therefore control for individual differences in familiarity with masked faces to avoid confounding effects on these outcome measures.

The fourth limitation pertains to the methodology of measuring interoceptive awareness. In the present study, interoceptive awareness was assessed using the MAIA, which primarily measures tendencies in daily contexts. However, such daily life tendencies do not necessarily reflect awareness triggered by salient bodily events. In our paradigm, facial stimuli elicited transient and salient external inputs, suggesting a potential mismatch between the context of interoceptive awareness and the demands of the task. Recent studies suggest that interoceptive awareness assessed in relation to salient events, such as through momentary experience sampling or experimental manipulations of bodily states, reflects aspects of awareness more directly tied to affective processing (Poerio et al., 2024; Wallman-Jones et al., 2022). In light of these considerations, the impact of emotional sensitivity on masked facial expressions might have been more apparent if interoceptive awareness was measured by questionnaires asking frequency of experiencing specific sensations rather than by assessing interoceptive awareness in daily life (Mandler et al., 1958; Porges, 1993). Furthermore, interoceptive awareness can also be assessed using objective and experimental indices targeting specific sensory modalities, beyond the self-reported tendencies measured by questionnaires. For example, cardiac interoceptive awareness has often been measured by the heartbeat detection tasks, and gastric interoceptive awareness has been measured by water-drinking or rectal distension (Garfinkel et al., 2015; van Dyck et al., 2016). Future research may therefore benefit from incorporating multiple methods to clarify which operationalization may predominantly enhance accuracy in emotion recognition of and/or emotional sensitivity to masked faces.

The fifth limitation is that the present study did not examine unconscious levels of emotional processing. Our methods focused exclusively on explicit behavioral measures, namely emotion categorization and ratings of valence and arousal, which do not reveal the processing stage at which interoceptive awareness exerts its influence. For example, it remains unclear whether higher scores on MAIA subscales such as emotional awareness and body-listening reflect stronger sensitivity to salient bodily signals themselves or differences in higher-level cognitive strategies for evaluating sensations and emotions. To address this issue, future research could combine behavioral tasks with physiological recordings of ongoing interoceptive signals during stimulus presentation, using techniques with high temporal resolution such as electroencephalography (EEG), electrocardiography (ECG), or electrogastrography (EGG). Previous studies have shown that heartbeat-evoked potentials modulate neural responses to emotional stimuli (e.g., Fukushima et al., 2011; Marshall et al., 2017), suggesting that such approaches may clarify the stage at which interoceptive and emotional processes interact, beyond what can be inferred from behavioral outcomes alone.

The sixth limitation concerns generalizability of the present findings to different populations. The present sample consisted exclusively of young adults from Japan, which constrains the extent to which the results can be generalized. Previous research suggests that impairments in recognizing masked facial emotions vary with age; for example, older adults exhibit lower accuracy than younger adults for anger, sadness, and fear, suggesting differential reliance on facial regions for emotion recognition (Slessor et al., 2022). With respect to interoception, developmental studies show that interoceptive awareness improves during childhood and approaches adult-like levels by late childhood or early adolescence (Koch & Pollatos, 2014; Maister et al., 2017). It appears relatively stable across early and middle adulthood, although evidence summarized by Murphy and Brewer (2024) indicates greater variability and potential decline in older age. Regarding cultural variation, studies of gaze allocation have demonstrated systematic differences in the use of facial cues during emotion recognition (e.g., Americans tend to rely more on the mouth region, whereas Japanese observers rely more on the eye region; Yuki et al., 2007; Blais et al., 2021; Hessels et al., 2025). In addition, cross-cultural studies report differences in interoceptive awareness as measured by both self-report and experimental tasks across diverse cultural groups, including Western, East Asian, and African samples (Ma-Kellams, 2014; Chentsova-Dutton & Dzokoto, 2014; Murphy & Brewer, 2024). Taken together, these findings suggest that the association between interoceptive awareness and performance on emotion recognition of masked faces in the present study may not generalize to populations differing in age or cultural background. Future studies should therefore extend investigations to participants across a broader age range and from diverse cultural contexts.

## 5. Conclusions

Results from the present study suggests that wearing a mask can reduce both accuracy and sensitivity of facial emotion recognition across multiple categories of emotions in the studied sample. Furthermore, results suggest that individuals with higher emotional awareness may perceive masked faces that expressed surprise as more positive than those with lower emotional awareness. These findings indicate that interoceptive awareness may play a compensatory role in emotion recognition when exteroceptive cues are limited, highlighting its potential importance for maintaining social communication under restricted facial visibility. Future research should replicate these findings under more controlled laboratory conditions with naturalistic stimuli, examine potential confounding variables, refine/reconsider approaches to measuring interoceptive awareness, and explore the generalizability of the present findings to broader populations.

## Figures and Tables

**Table 1:** Mean accuracy (% correct) in categorization of emotional facial expressions.

Subscale of MAIA	Interoceptive Awareness	Anger		Disgust		Fear		Happiness		Sadness		Surprise		Neutral	
		No-Mask	Mask	No-Mask	Mask	No-Mask	Mask	No-Mask	Mask	No-Mask	Mask	No-Mask	Mask	No-Mask	Mask
Noticing	Higher Group	71.32 (22.26)	68.63 (65.73)	60.54 (25.17)	51.47 (25.70)	21.32 (22.12)	11.28 (12.06)	97.55 (6.62)	95.83 (8.90)	65.44 (23.40)	50.25 (26.75)	93.63 (8.79)	75.74 (16.47)	93.63 (8.79)	75.74 (16.47)
	Lower Group	66.13 (25.04)	65.73 (28.86)	56.45 (25.18)	44.36 (25.17)	18.55 (20.88)	8.87 (15.26)	95.16 (14.32)	93.55 (16.73)	66.13 (22.41)	51.61 (25.36)	91.13 (16.83)	77.42 (21.51)	91.13 (16.83)	77.42 (21.51)
Not-distracting	Higher Group	69.74 (25.77)	68.42 (27.99)	56.91 (22.27)	53.29 (24.26)	18.42 (21.70)	13.16 (15.90)	96.38 (12.97)	94.74 (15.00)	67.76 (19.63)	51.97 (25.59)	90.79 (15.57)	72.70 (19.46)	90.79 (15.57)	72.70 (19.46)
	Lower Group	69.03 (21.31)	66.76 (21.39)	60.80 (27.44)	44.89 (26.32)	21.88 (21.59)	7.96 (10.12)	96.86 (7.20)	95.17 (9.80)	63.92 (25.46)	49.72 (26.75)	94.32 (8.72)	79.55 (17.07)	94.32 (8.72)	79.55 (17.07)
Attention Regulation	Higher Group	66.96 (23.40)	66.67 (25.10)	58.93 (24.74)	49.70 (23.67)	21.13 (21.82)	9.52 (9.49)	98.21 (6.52)	95.24 (9.94)	65.48 (23.07)	47.62 (27.92)	92.56 (9.59)	76.49 (17.51)	92.56 (9.59)	76.49 (17.51)
	Lower Group	71.88 (23.30)	68.44 (24.18)	59.06 (25.79)	47.81 (27.72)	19.38 (21.55)	11.25 (16.46)	95.00 (12.91)	94.69 (14.68)	65.94 (22.99)	54.06 (23.92)	92.81 (14.95)	76.25 (19.57)	92.81 (14.95)	76.25 (19.57)
Emotional Awareness	Higher Group	67.86 (25.60)	60.36 (28.52)	58.57 (26.74)	52.86 (24.65)	20.36 (23.89)	11.43 (12.99)	98.21 (5.38)	95.71 (7.39)	64.29 (23.71)	49.64 (27.20)	93.57 (8.23)	77.50 (16.27)	93.57 (8.23)	77.50 (16.27)
	Lower Group	70.48 (21.72)	72.87 (19.73)	59.31 (24.10)	45.75 (26.10)	20.21 (19.94)	9.57 (13.60)	95.48 (12.62)	94.42 (15.16)	66.76 (22.46)	51.60 (25.49)	92.02 (14.84)	75.53 (20.01)	92.02 (14.84)	75.53 (20.01)
Body-listening	Higher Group	69.29 (24.96)	62.50 (25.14)	58.97 (26.44)	52.45 (24.10)	17.40 (19.27)	17.39 (19.27)	97.01 (7.54)	94.57 (10.09)	62.77 (22.90)	45.38 (26.92)	93.21 (9.02)	76.36 (17.33)	93.21 (9.02)	76.36 (17.33)
	Lower Group	69.44 (21.43)	73.96 (22.44)	59.03 (23.64)	44.10 (26.97)	23.96 (23.98)	11.46 (16.47)	96.18 (12.96)	95.49 (14.99)	69.44 (22.65)	57.64 (23.58)	92.01 (15.85)	76.39 (20.00)	92.01 (15.85)	76.39 (20.00)
Trusting	Higher Group	66.29 (21.31)	66.29 (23.90)	56.82 (24.43)	51.90 (27.62)	21.59 (23.86)	9.09 (10.49)	98.49 (5.19)	95.83 (7.44)	70.08 (20.24)	54.92 (25.57)	93.94 (8.91)	76.14 (18.58)	93.94 (8.91)	76.14 (18.58)
	Lower Group	71.43 (24.61)	68.37 (25.14)	60.46 (25.69)	46.68 (24.18)	19.39 (20.10)	11.22 (14.93)	95.41 (12.42)	94.39 (14.89)	62.76 (24.27)	47.96 (26.31)	91.84 (14.33)	76.53 (18.51)	91.84 (14.33)	76.53 (18.51)

Standard deviations (*SD*s) of the mean are shown in parentheses.

**Table 2:** Mean rated scores of valence for the emotional facial expressions.

Subscale of MAIA	Interoceptive Awareness	Anger		Disgust		Fear		Happiness		Sadness		Surprise	
		No-Mask	Mask	No-Mask	Mask	No-Mask	Mask	No-Mask	Mask	No-Mask	Mask	No-Mask	Mask
Noticing	Higher Group	3.02 (0.70)	2.87 (0.63)	2.97 (0.70)	3.55 (0.85)	3.21 (0.61)	3.83 (0.70)	7.57 (1.10)	7.14 (0.81)	3.44 (0.65)	3.35 (0.54)	5.27 (0.71)	5.98 (0.89)
	Lower Group	3.11 (0.90)	3.06 (0.88)	3.09 (1.01)	3.59 (0.90)	3.38 (1.00)	3.94 (0.76)	7.32 (1.06)	6.97 (0.93)	3.48 (0.56)	3.57 (0.86)	5.18 (0.73)	4.98 (0.87)
Not-distracting	Higher Group	3.04 (0.79)	2.95 (0.78)	2.96 (0.90)	3.50 (0.89)	3.29 (0.82)	3.78 (0.68)	7.47 (0.91)	7.02 (0.87)	3.48 (0.56)	3.45 (0.74)	5.12 (0.74)	4.96 (0.80)
	Lower Group	3.06 (0.78)	2.93 (0.71)	3.07 (0.77)	3.62 (0.85)	3.26 (0.76)	3.95 (0.76)	7.48 (1.22)	7.13 (0.85)	3.43 (0.66)	3.42 (0.63)	5.33 (0.69)	5.12 (0.95)
Attention Regulation	Higher Group	3.24 (0.74)	3.06 (0.71)	3.13 (0.85)	3.64 (0.84)	3.38 (0.72)	3.95 (0.62)	7.29 (1.25)	6.92 (0.95)	3.33 (0.62)	3.53 (0.62)	5.17 (0.75)	5.05 (0.88)
	Lower Group	2.86 (0.78)	2.82 (0.76)	2.90 (0.79)	3.49 (0.90)	3.17 (0.84)	3.79 (0.82)	7.67 (0.84)	7.24 (0.72)	3.58 (0.58)	3.32 (0.73)	5.30 (0.68)	5.04 (0.89)
Emotional Awareness	Higher Group	3.11 (0.78)	2.96 (0.76)	3.06 (0.76)	3.63 (1.00)	3.41 (0.77)	3.97 (0.72)	7.75 (0.90)	7.17 (0.81)	3.45 (0.64)	3.43 (0.67)	5.19 (0.64)	5.27 (0.80)
	Lower Group	3.01 (0.78)	2.92 (0.73)	2.98 (0.89)	3.52 (0.76)	3.17 (0.78)	3.80 (0.72)	7.27 (1.17)	7.01 (0.89)	3.46 (0.59)	3.43 (0.70)	5.27 (0.77)	4.87 (0.91)
Body-listening	Higher Group	3.11 (0.74)	2.98 (0.64)	3.01 (0.75)	3.57 (0.80)	3.31 (0.69)	3.92 (0.70)	7.51 (1.17)	7.04 (0.93)	3.42 (0.62)	3.44 (0.58)	5.27 (0.69)	5.20 (0.76)
	Lower Group	2.98 (0.84)	2.88 (0.85)	3.02 (0.93)	3.56 (0.95)	3.23 (0.89)	3.82 (0.75)	7.43 (0.98)	7.13 (0.75)	3.50 (0.60)	3.41 (0.80)	5.19 (0.76)	4.84 (0.99)
Trusting	Higher Group	3.09 (0.74)	3.02 (0.64)	2.94 (0.75)	3.35 (0.75)	3.27 (0.80)	3.76 (0.68)	7.54 (1.12)	7.07 (0.91)	3.36 (0.53)	3.38 (0.62)	5.11 (0.73)	4.98 (0.88)
	Lower Group	3.02 (0.81)	2.89 (0.80)	3.07 (0.89)	3.71 (0.91)	3.28 (0.78)	3.95 (0.75)	7.43 (1.06)	7.08 (0.82)	3.52 (0.66)	3.46 (0.72)	5.32 (0.70)	5.09 (0.89)

Standard deviations (*SD*s) of the mean are shown in parentheses.

**Table 3:** Mean rated scores of arousal for the emotional facial expressions.

Subscale of MAIA	Interoceptive Awareness	Anger		Disgust		Fear		Happiness		Sadness		Surprise	
		No-Mask	Mask	No-Mask	Mask	No-Mask	Mask	No-Mask	Mask	No-Mask	Mask	NO-MASK	Mask
Noticing	Higher Group	6.80 (1.00)	6.79 (0.94)	6.24 (1.22)	6.24 (1.05)	6.80 (0.95)	6.77 (0.95)	6.79 (1.34)	6.47 (1.12)	5.23 (1.01)	5.53 (1.19)	5.27 (0.71)	5.08 (0.89)
	Lower Group	6.53 (1.09)	6.38 (0.89)	5.89 (1.34)	5.99 (1.07)	6.50 (1.03)	6.21 (1.04)	6.59 (1.12)	6.26 (0.99)	5.03 (1.22)	5.11 (1.04)	5.18 (0.73)	4.98 (0.87)
Not-distracting	Higher Group	6.74 (1.10)	6.57 (0.98)	6.01 (1.40)	5.90 (1.16)	6.67 (1.07)	6.45 (1.13)	6.50 (1.41)	6.19 (118)	5.05 (1.21)	5.33 (1.15)	5.12 (0.74)	4.96 (0.80)
	Lower Group	6.66 (0.99)	6.69 (0.90)	6.20 (1.15)	6.36 (0.92)	6.70 (0.92)	6.65 (0.90)	6.90 (1.10)	6.56 (0.96)	5.24 (0.99)	5.41 (1.15)	5.33 (0.69)	5.12 (0.95)
Attention Regulation	Higher Group	6.25 (1.05)	6.41 (0.97)	5.97 (1.26)	5.92 (1.10)	6.46 (1.03)	6.40 (0.98)	6.46 (1.39)	6.21 (1.16)	5.21 (1.18)	5.24 (1.10)	5.17 (0.75)	5.05 (0.88)
	Lower Group	6.89 (1.00)	6.86 (0.85)	6.27 (1.27)	6.39 (0.97)	6.92 (0.89)	6.72 (1.03)	6.98 (1.06)	6.58 (0.96)	5.09 (1.00)	5.51 (1.20)	5.30 (0.68)	5.04 (0.89)
Emotional Awareness	Higher Group	6.67 (0.92)	6.66 (0.84)	6.08 (1.19)	6.11 (1.14)	6.67 (0.87)	6.28 (1.24)	6.78 (1.49)	6.28 (1.24)	5.22 (0.96)	5.26 (1.11)	5.27 (0.80)	5.27 (0.80)
	Lower Group	6.72 (1.12)	6.61 (1.01)	6.13 (1.33)	6.17 (1.01)	6.71 (1.08)	6.47 (0.94)	6.67 (1.07)	6.47 (0.94)	5.10 (1.19)	5.46 (1.17)	4.87 (0.91)	4.87 (0.91)
Body-listening	Higher Group	6.71 (1.03)	6.56 (0.98)	6.07 (1.26)	6.05 (1.05)	6.70 (1.06)	6.58 (1.02)	6.72 (1.41)	6.32 (1.19)	5.18 (1.12)	5.42 (1.16)	5.27 (0.69)	5.20 (0.76)
	Lower Group	6.69 (1.06)	6.72 (0.89)	6.16 (1.29)	6.27 (1.07)	6.67 (0.91)	6.53 (1.02)	6.71 (1.05)	6.48 (0.91)	5.12 (1.07)	5.32 (1.15)	5.19 (0.76)	4.84 (0.99)
Trusting	Higher Group	6.64 (1.00)	6.45 (0.91)	5.88 (1.19)	5.99 (1.06)	6.55 (0.97)	6.43 (0.98)	6.81 (1.24)	6.35 (0.98)	4.99 (1.21)	5.11 (1.02)	5.11 (0.73)	4.98 (0.88)
	Lower Group	6.74 (1.07)	6.75 (0.94)	6.27 (1.30)	6.25 (1.05)	6.78 (1.00)	6.65 (1.03)	6.65 (1.28)	6.44 (1.15)	5.26 (1.01)	5.55 (1.20)	5.32 (0.70)	5.09 (0.89)

Standard deviations (*SD*s) of the mean are shown in parentheses.

**Table 4:** Results of three-way ANOVAs for accuracy of categorization of emotions.

Subscale of MAIA	Main Effect: Presence of Mask		Main Effect: Emotion		Main Effect: Interoceptive Awareness		Interaction: Presence of Mask × Emotion		Interaction: Presence of Mask × Interoceptive Awareness		Interaction: Emotion × Interoceptive Awareness		Interaction: Presence of Mask × Emotion × Interoceptive Awareness	
	*F*_1, 80_	*ηp* ^2^	*F*_6, 480_	*ηp* ^2^	*F*_1, 80_	*ηp* ^2^	*F*_6, 480_	*ηp* ^2^	*F*_1, 80_	*ηp* ^2^	*F*_6, 480_	*ηp* ^2^	*F*_6, 480_	*ηp* ^2^
Noticing	96.896 ***	0.548	184.085 ***	0.697	0.376	0.005	13.140 ***	0.141	0.028	<0.001	0.809	0.010	0.406	0.005
Not-distracting	106.530 ***	0.571	190.851 ***	0.705	0.004	<0.001	14.898 ***	0.157	5.206 *	0.061	0.538	0.007	2.131	0.026
Attention Regulation	103.737 ***	0.565	191.863 ***	0.706	0.109	0.0014	14.493 ***	0.153	0.072	<0.001	0.265	0.003	0.885	0.011
Emotional Awareness	101.440 ***	0.559	189.703 ***	0.703	0.0003	<0.001	13.972 ***	0.149	0.004	<0.001	0.895	0.011	1.773	0.022
Body-listening	101.759 ***	0.560	190.628 ***	0.704	0.892	0.011	14.825 ***	0.156	0.062	<0.001	1.494	0.018	2.966 *	0.036
Trusting	97.836 ***	0.550	187.234 ***	0.701	0.987	0.012	14.641 ***	0.155	0.453	0.006	0.856	0.011	1.276	0.016

* *p* < 0.05, *** *p* < 0.001.

**Table 5:** Results of three-way ANOVAs for the rating scores of valence.

Subscale of MAIA	Main Effect: Presence of Mask		Main Effect: Emotion		Main Effect: Interoceptive Awareness		Interaction: Presence of Mask × Emotion		Interaction: Presence of Mask × Interoceptive Awareness		Interaction: Emotion × Interoceptive Awareness		Interaction: Presence of Mask × Emotion × Interoceptive Awareness	
	*F*_1, 80_	*ηp* ^2^	*F*_5, 400_	*ηp* ^2^	*F*_1, 80_	*ηp* ^2^	*F*_5, 400_	*ηp* ^2^	*F*_1, 80_	*ηp* ^2^	*F*_5, 400_	*ηp* ^2^	*F*_5, 400_	*ηp* ^2^
Noticing	5.487 *	0.064	480.767 ***	0.857	0.095	0.001	26.938 ***	0.252	0.301	0.004	0.999	0.012	0.447	0.006
Not-distracting	4.951 *	0.058	514.413 ***	0.865	0.479	0.006	29.405 ***	0.269	0.432	0.005	0.306	0.004	0.406	0.005
Attention Regulation	5.119 *	0.060	535.225 ***	0.870	0.204	0.003	30.585 ***	0.277	1.070	0.013	2.797	0.034	2.207	0.027
Emotional Awareness	5.223 *	0.061	513.054 ***	0.865	2.265	0.028	30.231 ***	0.274	0.060	<0.001	0.640	0.008	3.036 *	0.037
Body-listening	4.788 *	0.056	510.822 ***	0.865	0.527	0.007	29.488 ***	0.269	0.361	0.004	0.425	0.005	0.990	0.012
Trusting	4.350 *	0.052	503.189 ***	0.863	0.683	0.008	27.557 ***	0.256	0.609	0.008	0.784	0.010	0.918	0.011

* *p* < 0.05, *** *p* < 0.001.

**Table 6:** Results from three-way ANOVAs for the rating scores of arousal.

Subscale of MAIA	Main Effect: Presence of Mask		Main Effect: Emotion		Main Effect: Interoceptive Awareness		Interaction: Presence of Mask × Emotion		Interaction: Presence of Mask × Interoceptive Awareness		Interaction: Emotion × Interoceptive Awareness		Interaction: Presence of Mask × Emotion × Interoceptive Awareness	
	*F*_1, 80_	*ηp* ^2^	*F*_5, 400_	*ηp* ^2^	*F*_1, 80_	*ηp* ^2^	*F*_5, 400_	*ηp* ^2^	*F*_1, 80_	*ηp* ^2^	*F*_5, 400_	*ηp* ^2^	*F*_5, 400_	*ηp* ^2^
Noticing	5.615 *	0.066	61.638 ***	0.435	3.489	0.042	4.028 ***	0.048	1.440	0.018	0.435	0.005	0.566	0.007
Not-distracting	4.863 *	0.057	66.855 ***	0.455	1.727	0.021	4.486 **	0.053	1.042	0.003	0.656	0.008	0.758	0.009
Attention Regulation	4.463 *	0.048	67.979 ***	0.459	4.072 *	0.048	4.675 **	0.055	0.279	0.003	1.095	0.014	1.558	0.019
Emotional Awareness	4.512 *	0.053	64.830 ***	0.448	0.001	<0.001	4.319 **	0.051	0.027	<0.001	0.232	0.003	2.804 *	0.034
Body-listening	4.272 *	0.051	67.390 ***	0.457	0.004	<0.001	4.460 **	0.053	0.115	0.0014	0.646	0.008	0.974	0.012
Trusting	4.987 *	0.059	65.735 ***	0.451	1.795	0.022	4.399 **	0.052	0.548	0.007	0.711	0.009	0.805	0.010

* *p* < 0.05, ** *p* < 0.01, *** *p* < 0.001.

## Data Availability

The datasets used and analyzed during the study are available from the corresponding author upon reasonable request.
